# Design and Manufacturing of a Novel Trabecular Tibial Implant

**DOI:** 10.3390/ma16134720

**Published:** 2023-06-29

**Authors:** Yongdi Zhang, Baoyu Sun, Lisong Zhao, Guang Yang

**Affiliations:** College of Mechanical Engineering, Hebei University of Science and Technology, Shijiazhuang 050018, China; sunbaoyu0105@163.com (B.S.); ls_z0212@163.com (L.Z.)

**Keywords:** tibial implant, porous structure, gradient porosity, titanium alloy, selective laser melting

## Abstract

The elastic modulus of traditional solid titanium alloy tibial implants is much higher than that of human bones, which can cause stress shielding. Designing them as a porous structure to form a bone-like trabecular structure effectively reduces stress shielding. However, the actual loading conditions of bones in different parts of the human body have not been considered for some trabecular structures, and their mechanical properties have not been considered concerning the personalized differences of other patients. Therefore, based on the elastic modulus of the tibial stem obtained from Quantitative Computed Tomography (QCT) imaging between 3.031 and10.528 GPa, and the load-bearing state of the tibia at the knee joint, a porous structure was designed under compressive and shear loading modes using topology optimization. Through comprehensive analysis of the mechanical and permeability properties of the porous structure, the results show that the Topology Optimization–Shear-2 (TO-S2) structure has the best compressive, shear mechanical properties and permeability and is suitable as a trabecular structure for tibial implants. The Gibson–Ashby model was established to control the mechanical properties of porous titanium alloy. A gradient filling of porous titanium alloy with a strut diameter of 0.106–0.202 mm was performed on the tibial stem based on the elastic modulus range, achieving precise matching of the mechanical properties of tibial implants and closer to the natural structure than uniformly distributed porous structures in human bones. Finally, the new tibial implant was printed by selective laser melting (SLM), and the molding effect was excellent.

## 1. Introduction

Titanium and its alloys are suitable for orthopedic implants due to their excellent fatigue strength, biocompatibility, corrosion resistance, and plasticity. Most traditional titanium alloy implants are dense Ti6Al4V alloys with an elastic modulus of 113 GPa, while the elastic modulus of human cortical bone is generally between 2 and 20 GPa [1], resulting in stress shielding between the implant and human bone [2]. According to Wolff’s law, stress shielding reduces bone stimulation, causes cortical bone thinning, and leads to aseptic implant loosening [3]. In recent years, porous structures have received widespread attention due to their low density, large specific surface area, high strength, and good energy absorption properties [4,5]. Traditional methods such as powder metallurgy, powder foaming, and fiber sintering can produce porous titanium alloys. Still, uneven pore distribution and blockage are common, making it challenging to meet the needs of the medical field. The emergence of SLM additive manufacturing technology can achieve the formation of arbitrarily complex porous implants with accurate shape control and is commonly used in the medical field to prepare porous titanium alloys that mimic the trabecular structure of human bone [6].

Zhang et al. [7] designed personalized tibial implants through reverse and forward methods combined with the finite element method. Compared with traditional implants, the stress distribution is more uniform, but the porous structure is not used to simulate the human trabecular bone, and its elastic modulus is relatively high. Most studies on porous structures aim to simulate bone structure by changing their mechanical properties, such as elastic modulus and compressive strength [8]. Porous implants need to meet the bone ingrowth requirements, and the main factors affecting bone ingrowth are porosity, pore size, and pore shape. Porous structures with appropriate pore size and porosity provide enough space for cell proliferation [9], and different porosity and pore shapes can lead to differences in permeability and bone ingrowth effect [10]. Chakkkravarthy et al. [11] prepared porous scaffolds using SLM technology and quantitatively evaluated the implant-induced biological activity and Young’s modulus. It was found that fibroblasts have superior cell motility, protein binding ability and adhesion in porous scaffolds, and they show excellent ability to prevent postoperative tumor recurrence. More interestingly, the printed scaffold almost matches Young’s modulus of the human skeleton.

In addition, various skeletal sites have different mechanical requirements [12,13], and the spine is often subjected to compression and bending loads during exercise [14]. At the same time, the tibia of the knee joint bears not only external pressure load but also specific shear loads [15,16]. However, most of the current research related to topology optimization of porous structures is based on compressive loads, and tibial implants also need to withstand large shear loads. Topology optimization design of porous structures under shear loads is of great significance for porous tibial implants to achieve performance matching with human bones.

Therefore, this article aims to study bone microstructure-matched tibial implants and design a porous structure that matches the deformation and stress conditions according to the load-bearing requirements of the tibial plateau. The porous titanium alloy is precisely regulated and gradient-filled based on the elastic modulus of bone. The designed bone microstructure-matched tibial implant can select the appropriate porous structure according to the stress conditions of the bone, further improving the degree of personalization of the implant and achieving precise matching with the mechanical properties of the human bone.

## 2. Personalized Design of Tibial Implants

QCT is an image-based bone mechanical property analysis method [17]. It mainly uses a calibration phantom to convert the gray value in the CT image into an equivalent volume of bone density to evaluate the bone density distribution of different bone sites. It is not affected by the patient’s factors. The bone mineral density of the patient’s bone is different, and the corresponding elastic modulus is also different. It can more accurately measure the density value of cortical bone and cancellous bone so that the elastic modulus of the porous structure can be accurately matched with the elastic modulus of the corresponding part of the bone. Therefore, the evaluation of bone mechanical properties based on QCT images is often used to predict the mechanical properties of the human lumbar spine and tibia and determine the corresponding treatment plan.

### 2.1. Establishment of Tibial Implant Model

**Figure 1:**
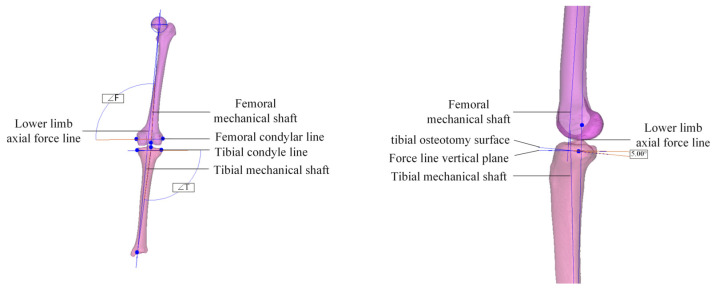
Schematic diagram of landmark points for osteotomy.

The knee joint bones were scanned using QCT to collect DICOM format 2D sectional scan data, which were imported into medical image processing software Mimics 21.0 for processing and conversion into a Stereolithography (STL) model. STL file is a format that expresses the structure of the real model through the combination of many small triangular facets. 3-Matic 11.0 software was used to perform standardized simulated osteotomy on the tibia to obtain a personalized tibial implant. The knee joint bone STL model was imported into 3-Matic software, and the following marks were made [16], as shown in Figure 1: the connection between the medial and lateral condyles of the femur is the femoral condyle line; the connection between the medial and lateral tibial plateaus is the tibial plateau line; the center point of the femoral head and the center point of the distal end of the femur are connected to form the femoral mechanical axis (FMA); the center point of the upper border of the tibial plateau and the center point of the distal end of the tibia are connected to form the tibial mechanical axis (TMA); the angle between the femoral mechanical axis and the femoral condyle line is ∠F between 97° and 99°; and the angle between the tibial mechanical axis and the tibial plateau line is ∠T between 87° and 90°. Due to a certain degree of varus angle between the femoral and tibial mechanical axes, the osteotomy plane was installed and positioned with a flip angle of less than 5° according to the experience of knee replacement surgery research [18].

The fitting degree between implants and the knee joint after the osteotomy is crucial to postoperative rehabilitation. Therefore, measurements of the morphological parameters of the tibia in the knee joint are taken. Based on the morphological indicators of the proximal tibia [19], geometric morphological parameters of the simulated osteotomized tibia are measured in 3-Matic software.

**Figure 2:**
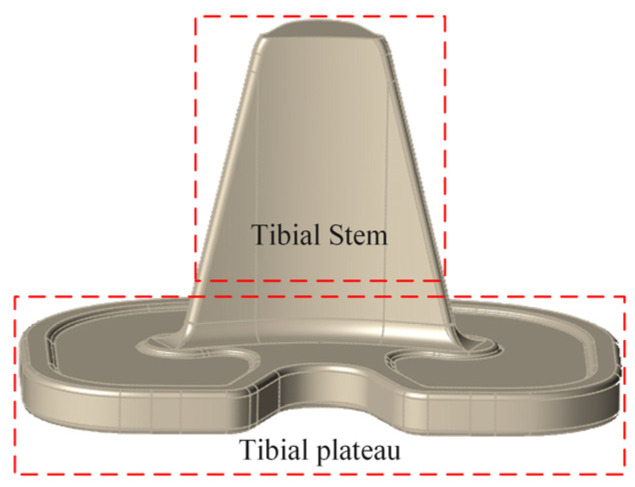
Personalized tibial implant model.

The personalized tibial implant was designed using the three-dimensional forward design software Solidworks 2021. The main parameters of the model were determined based on the measurement results of the tibial geometry. A solid model of the tibial implant that conforms to the patient’s knee joint anatomy was designed, as shown in Figure 2. The tibial implant is an essential part of total knee arthroplasty, consisting of a tibial tray and a tibial stem implanted into the tibial bone after osteotomy. The dense titanium alloy has a significant difference in elastic modulus compared to the host bone tissue, which can easily cause a stress-shielding effect. Furthermore, the weight difference between the implant and normal bone may cause discomfort to the patient. Therefore, it is necessary to design bone trabeculae in the tibial stem region of personalized tibial implants to meet the mechanical properties of the host bone tissue while achieving lightweight effects.

### 2.2. Tibia Implant Trabecular Region Segmentation

Based on the measurement of bone density from tibial skeletal CT images, it is known through experience that cortical bone density is relatively high in the bone shaft, mainly used for supporting body load, while the bone density at the metaphysis is relatively low, mainly used for forming joints and bone growth. The CT grayscale values of the tibia increase gradually from the proximal end to the distal end, indicating that the threshold of the tibial skeleton shows a gradient-increasing trend from the proximal end to the distal end [20].

In Mimics software, CT images of the partially resected tibia after osteotomy can be obtained through a 3D solid model. Since the loading on the human knee joint is mainly axial, cross-sections are primarily used for measuring bone mineral density in the ROI region. The threshold of the pixel area where the tibial proximal cortical bone mask is located is exported, and the average threshold of the cortical bone mask of each CT image is calculated based on the threshold data of the CT image. The threshold range of normal cortical bone in the human body is 300–1160 HU [21]. To divide cortical bone with different threshold ranges, a QCT value change of 100 HU is used as the threshold interval, with an error of no more than 0.1 g/cm^3^. A layer of the bone trabecula is formed when the threshold value changes over 100 HU from the start to the end threshold value.

**Figure 3:**
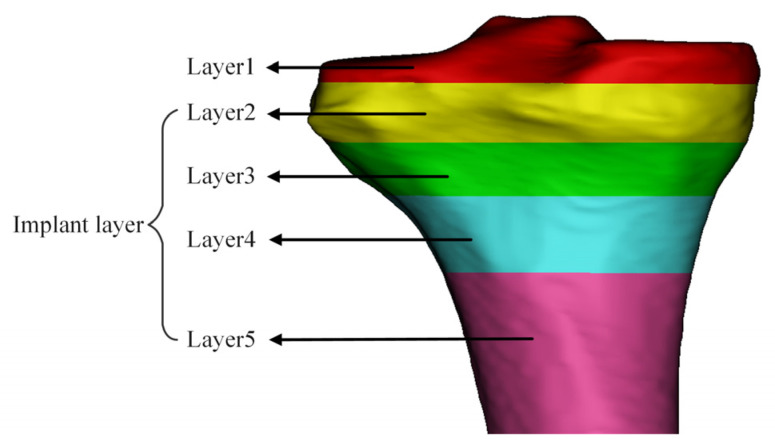
Schematic diagram of tibial trabecular bone region partitioning.

The tibial structure is segmented into multiple trabecular layers with masks of different colors based on different threshold intervals, and the starting and ending thresholds of each layer are recorded. As shown in Figure 3, the threshold interval of the tibia is divided into five layers, Layer 1 is the cross-section layer, and Layer 2 to Layer 5 are the layers where prostheses can be implanted. The trabecular layers in different threshold intervals have different apparent densities. The evident density values corresponding to the starting and ending thresholds of various trabecular layers are calculated by Equations (1) and (2) [22]. The elastic modulus E related to the apparent density value *ρ* is calculated by Equations (3)–(6) [23], thus effectively evaluating the mechanical properties of different trabecular layers. The characterization results are shown in Table 1, and the obtained elastic modulus is consistent with the elastic modulus of human cortical bone [1].

(1) $$\rho =1.9\times 10^{-3}QCT+0.105\left(QCT<816\right)$$

(2) $$\rho =7.69\times 10^{-4}QCT+1.028\left(QCT\geq 816\right)$$

(3) $$E=1007\rho_{1}{}^{2}\left(\rho \leq 0.25\right)$$

(4) $$E=255\rho_{2}(0.25<\rho_{2}\leq 0.4)$$

(5) $$E=2972\rho_{3}{}^{2}-933\rho_{3}\left(0.4<\rho_{3}\leq 1.2\right)$$

(6) $$E=1763\rho_{4}{}^{3.25}\left(\rho_{4}>1.2\right)$$

**Figure 4:**
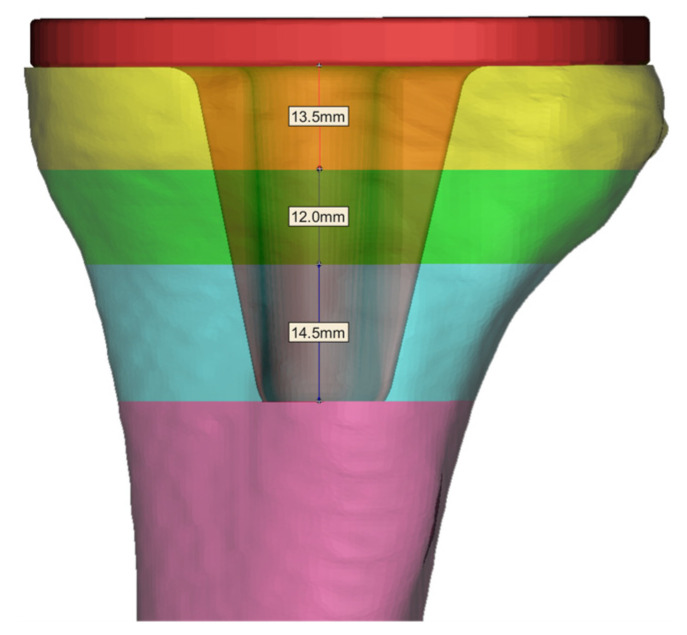
Division of tibial plateau subchondral bone into trabecular layers and their thickness.

According to the tibial trabecular layer zoning diagram, Mimics software was used to simulate the tibial implant placed in the tibial region. The results showed that Layer 2 to Layer 4 were the implantation sites of the tibial stem of the implant, and the required elastic modulus of the tibial stem trabecular layer was 3.031–10.528 GPa. The thickness of each trabecular layer required for the zone was measured, as shown in Figure 4.

## 3. Topology Optimization Design and Performance Research of Unit Cell Structures

### 3.1. Topological Optimization Design for Unit Cells

**Figure 5:**
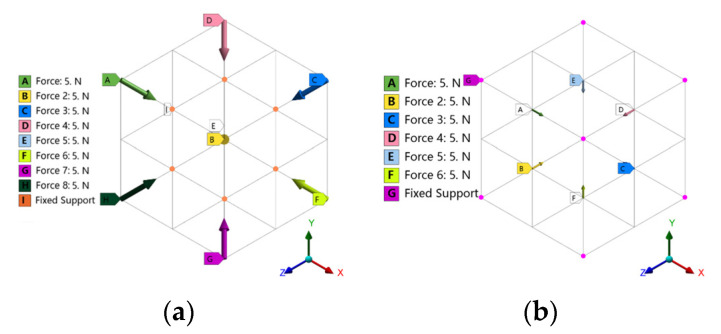
Compressed load condition: (**a**) Vertex load; (**b**) Face center load.

Compressed loads can be divided into four main types in the working conditions shown in Figure 5. The first type is that the eight vertices are subjected to compressed loads (with a magnitude of 5N and a direction pointing to the center of the cube), and six face centers are fixed constraints. The second type is that the six face centers are subjected to compressed loads (with a magnitude of 5N and a direction perpendicular to the plane), and the eight vertices are fixed constraints.

**Figure 6:**
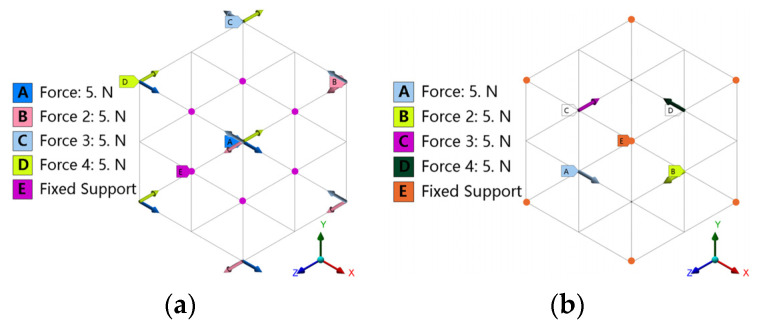
Shear load condition: (**a**) vertex load; (**b**) face center load.

Shear loads can mainly be divided into four types of conditions, as shown in Figure 6. The first type is that the four vertices of two faces in the X+ and Z+ directions are subjected to forces perpendicular to the intersection line of the two faces with a magnitude of 5N. The X- and Z- directions are the same, and the six face centers are subjected to fixed constraints. The second type is that the two face centers in the X+ and Z+ directions are subjected to forces perpendicular to the intersection line of the two faces with a magnitude of 5N. The X- and Z- directions are the same, and the eight vertices are subjected to fixed constraints.

**Figure 7:**
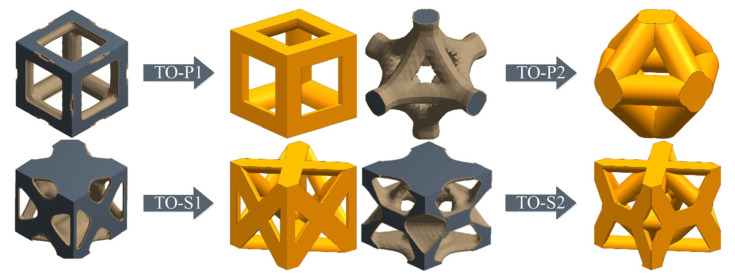
Reconstruction of topological cell models.

Using the CAD design method and three-dimensional modeling software UG 12.0, a simple supporting strut was established, and after various logical operations were performed for topological optimization, a unit cell was reconfigured. The type and abbreviation of porous structure in topology optimization are shown in Table 2, and the structure types are shown in Figure 7.

The porosity of porous structure can be calculated using the following formula.

(7) $$P=\left(1-\frac{V}{V_{S}}\right)\times 100\%$$

where *P*, *V* and *V_s_* represent the porosity, the volume of the unit cell structure and the volume of the maximum peripheral boundary, respectively.

**Figure 8:**
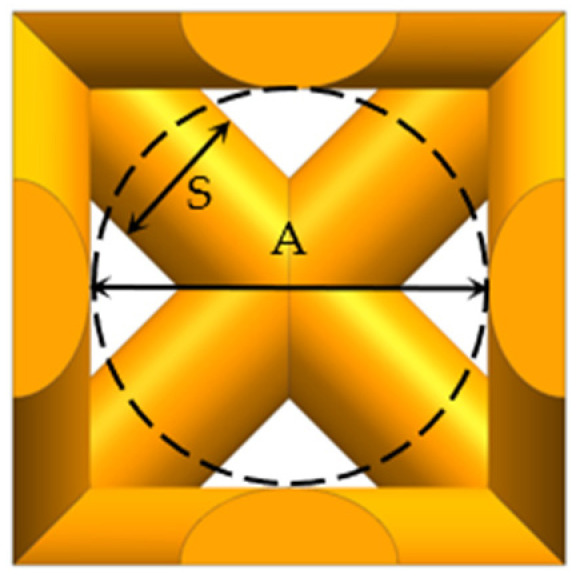
Schematic diagram of structural geometric parameters.

Figure 8 is a schematic diagram of the geometric parameters of the unit cell structure (S is the strut diameter, and A is the pore diameter).

Four kinds of unit cell structures of 1 mm × 1 mm × 1 mm were designed, with a porosity of 60–90% and a pore size of about 400–800 μm, which can meet the conditions for human bone tissue to grow in [24]. The porosity and pore diameter are controlled by changing the diameter of the strut. The geometric parameters of different types of topological units are shown in Table 3.

**Figure 9:**
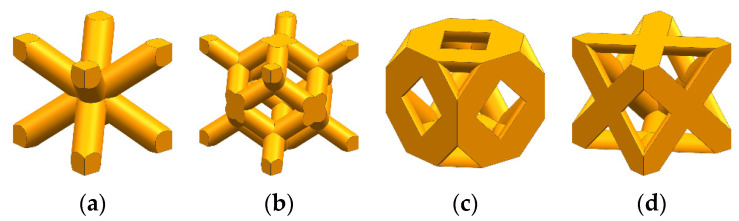
Four common types of basic unit cell structures: (**a**) BCC, (**b**) RDC, (**c**) DCC, (**d**) FCC.

In addition to the four cell structures generated from topological optimization, four common basic cell structures [25,26,27,28], including Body-Centered Cubic (BCC), Rhombic Dodecahedron Cubic (RDC), Diagonal Centered Cubic (DCC), and Face-Centered Cubic (FCC) were also selected for comparative study to investigate the mechanical properties of the topologically optimized cells of distinct structural types, as shown in Figure 9. According to the needs, the size models of four kinds of ordinary basic unit cell structures under various porosity are constructed, and the geometric parameters are shown in Table 4.

The mathematical model for the strut diameter S and porosity P in the table above was fitted by a quadratic polynomial. The fitting equation is y = A + Bx + Cx^2^, where y represents the porosity P, and x represents the strut diameter S. The strut diameter-porosity fitting equations of eight types of unit cell structures are shown in Table 5. The correlation coefficient R2 is above 0.99 for all kinds, and the fitting results are highly correlated, achieving controllability of the unit cell structure model.

### 3.2. Mechanical Performance Analysis

The mechanical properties of materials refer to the mechanical characteristics of materials under various external loads such as tension, compression, shear, bending and torsion. The mechanical properties studied in this paper are the compression and shear properties of porous structures.

**Figure 10:**
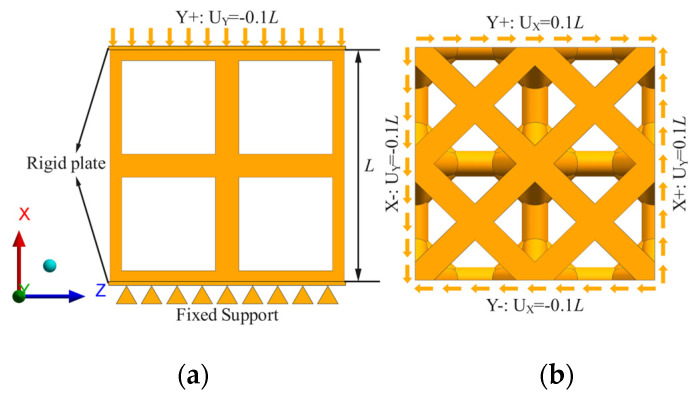
Load boundary conditions: (**a**) compression load, (**b**) shear load.

Using ANSYS Workbench 19.2 software to perform static compression and shear simulations on eight porous structures (TO-P1, TO-P2, TO-S1, TO-S2, BCC, RDC, DCC, FCC), respectively, the material properties are Ti6Al4V alloy, as shown in Table 6. Proximity and curvature function is used for mesh generation, and the optimal mesh size is 0.05 mm. The boundary conditions for the porous structures were set under axial compression, as shown in Figure 10a. The boundary conditions for the porous structures were set under pure shear, as shown in Figure 10b.

### 3.3. Permeability Analysis

The specific surface area of a porous structure is the ratio of the internal surface area (the surface area inside the wall of the porous structure) to the total volume (the sum of the solid volume and the pore volume), as shown in Equation (8).

(8) $$\delta =\frac{S}{V}$$

**Figure 11:**
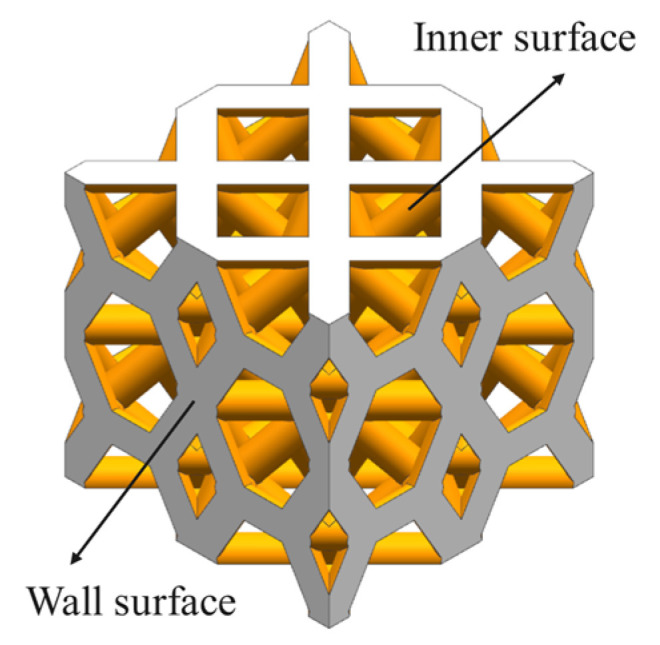
Schematic diagram of specific surface area of porous structure.

In the equation, *δ* represents the specific surface area of the porous structure, *S* represents the internal surface area of the porous structure, and *V* represents the total volume of the porous structure. The schematic diagram of the specific surface area of porous structures is shown in Figure 11.

The hydraulic conductivity coefficient K of a porous structure is an intrinsic property of the structure. It can be calculated using Darcy’s law with Equation (9):

(9) $$K=\frac{Q\mu L}{A\Delta P}$$

In the equation, *K* is the hydraulic conductivity coefficient, *Q* is the volumetric flow rate (m^3^/s), *µ* is the fluid viscosity coefficient (kg/m/s), *L* is the distance between the inlet and outlet (m), *A* is the inlet cross-sectional area (m^2^), and ∆*P* is the pressure drop between the inlet and outlet (MPa).

**Figure 12:**
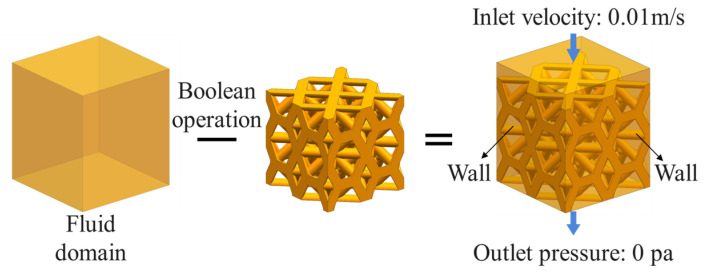
Modeling of the seepage flow and setting of boundary conditions.

This passage describes the simulation of flow processes through different porous structures using ANSYS Fluent 19.2 software, taking TO-S2 as an example. The modeling of the flow model and the boundary conditions are shown in Figure 12. First, the fluid domain model is created, and Boolean operations are performed on the porous structure model to obtain the fluid region of the porous structure. To avoid boundary effects caused by the inlet area, a virtual fluid region is reserved at the upper end of the fluid domain. Since the liquid in the human body is in a static pressure state and flows very slowly, laminar flow is selected as the flow performance state [29].

## 4. Results and Discussion

### 4.1. Mechanical Performance

**Figure 13:**
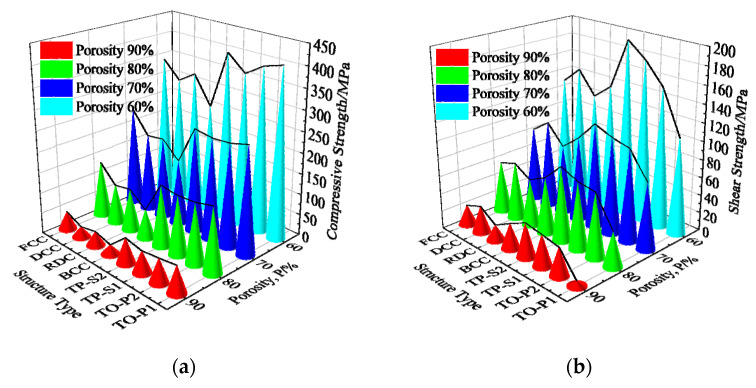
Mechanical performance simulation results: (**a**) Compressive strength; (**b**) Shear strength.

Compression and shear simulation analyses were performed on eight types of porous structures of titanium alloys ranging from 60% to 90% porosity. A three-dimensional line graph was plotted to visually represent the compressive and shear performance of different types of porous structures, as shown in Figure 13. In the compression simulation analysis, TO-P1, TO-S2, and TO-P2 have relatively high compressive strength, while RDC, DCC, and BCC have relatively low compressive strength, and FCC and TO-S1 are moderate. Among the eight structures, TO-P1, TO-S2, and TO-P2 have better compressive performance. In the shear simulation analysis, TO-S2 and TO-S1 have the highest shear strength, while TO-P2, DCC, BCC, and FCC are moderate, and RDC and TO-P1 have relatively low shear strength. Among the eight structures, TO-S2 and TO-S1 have better shear performance. The results show that the four porous structures based on topological optimization exhibit superior mechanical behavior.

**Figure 14:**
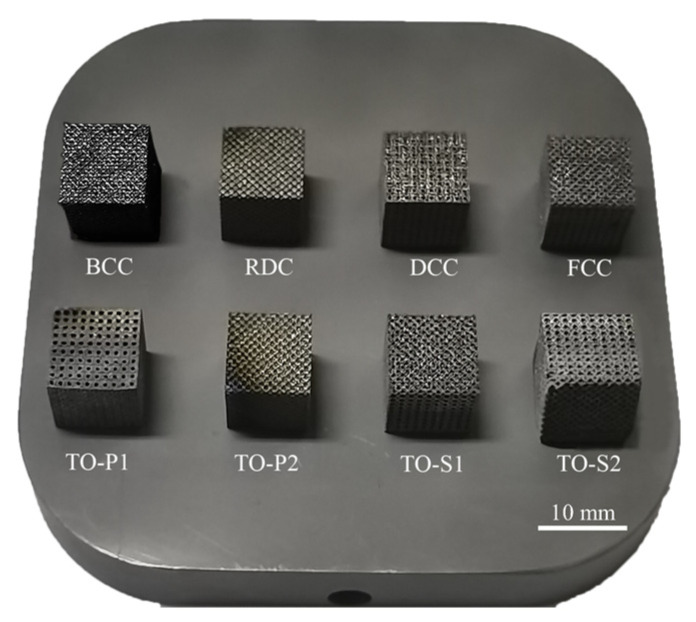
Porous structure compression specimen.

Using a RENISHAW AM250 printer to prepare eight types of porous Ti6Al4V compression specimens, with three specimens per type, the compression specimens were formed by arranging 10 × 10 × 10 unit cells in a 3D array. The processing parameters were laser power of 200 W, scanning speed of 1200 mm/s, scanning spacing of 140 μm, and powder layer thickness of 30 μm, with 99.99% pure argon gas filled in the forming chamber. The morphology of the porous structure is shown in Figure 14. 

**Figure 15:**
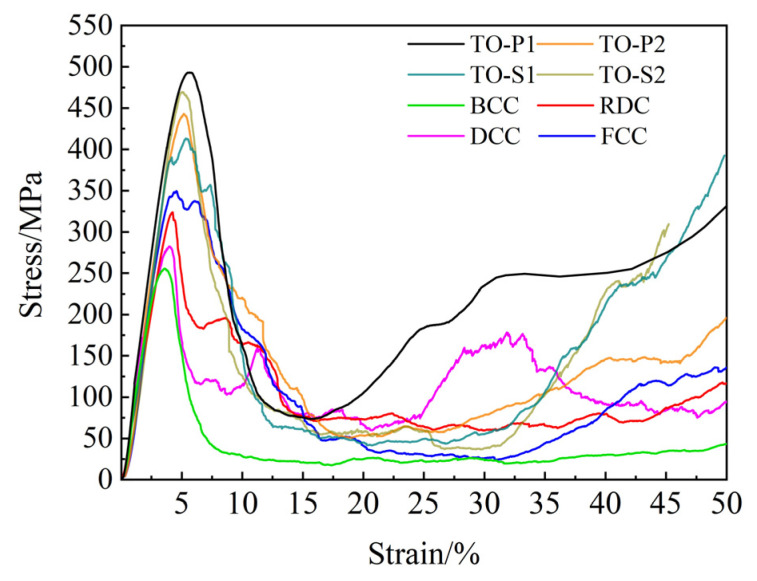
Compression stress–strain curves for eight porous Ti6Al4V specimens.

**Figure 16:**
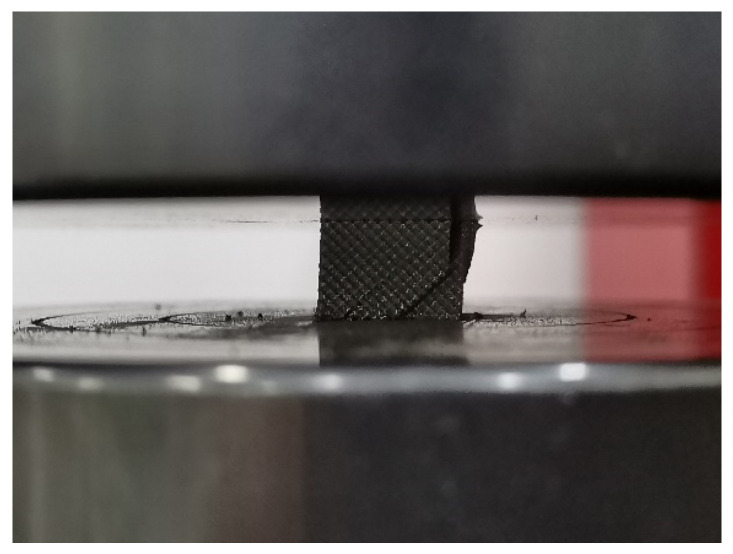
Compression failure of porous specimen.

After forming, the compression performance of the eight specimens was tested using a CMT5105 electronic universal testing machine, with a compression speed of 1 mm/min, and each type of specimen was tested three times with the average taken. After the compression experiments were completed, the raw data were exported, and the compression stress–strain curves of the porous Ti6Al4V specimens were plotted in Origin 2018 software, as shown in Figure 15. Additionally, the compression failure diagram of the porous structure is shown in Figure 16.

It can be seen from the compression stress–strain curve that the compression process of porous titanium alloy can be divided into three stages as a whole. The first stage is the linear elastic stage. In this stage, the strut undergoes elastic deformation, and the shape of the stress–strain curve is an inclined straight line. As the specimen is continuously compressed to reach the limit of elastic deformation, the slope of the curve changes and the stress reaches the maximum value. The second stage is the stress platform stage. In this stage, the struts begin to yield, the pores between the structures are continuously compressed, and the specimens are destroyed layer by layer. With the increase of strain, the stress value fluctuates, but the growth is not large. The third stage is the densification stage. In this stage, the struts are compressed to contact each other, the pores are compressed, and the specimens are gradually densified. At this time, the stress value increases rapidly with the increase of strain. It can be seen from Figure 15 that the compressive strength of the topologically optimized porous structure is higher than 400 MPa, which is significantly higher than the other four ordinary porous structures. 

It can be seen from the above analysis that the mechanical properties of the Ti6Al4V porous structure based on topology optimization are obviously better than those of the other four ordinary porous structures. The experimental results are consistent with the simulation results, which proves the reliability of the compression performance simulation analysis.

### 4.2. Permeability

The specific surface area of four types of porous structures was calculated and shown in Table 7. The table shows that TO-S2 has the highest specific surface area among its permeability properties, which allows for better cell adhesion and proliferation. 

The simulation results for permeability are shown in Table 8. Although its permeability coefficient is not the highest, it meets the permeability requirements (0.027 − 20.0 × 10^−9^ m^2^) for use as a human bone implant.

**Figure 17:**
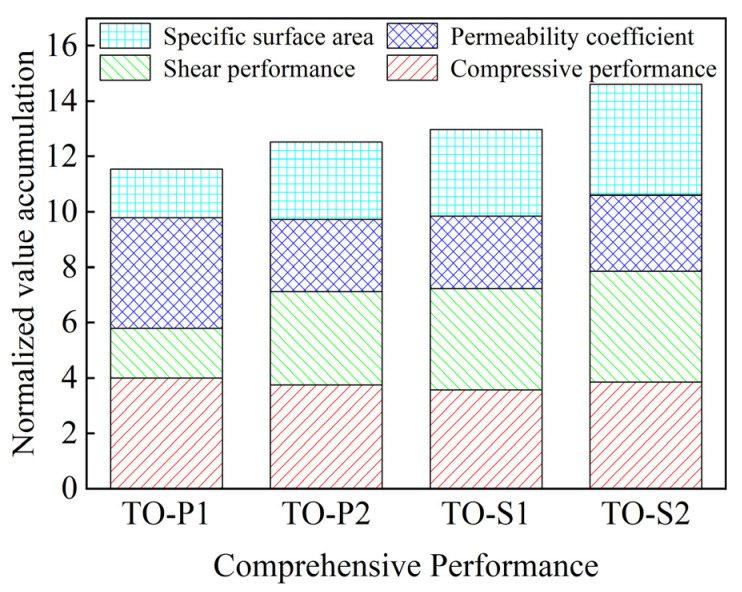
Comprehensive performance normalized superposition results.

Figure 17 shows the normalized performance of porous structures with four different porosities (actual value divided by the maximum value for the corresponding structure) superimposed. The maximum normalized value for each performance is 4. The permeability of the TO-S2 structure is similar to that of TO-P2 and TO-S1, but its specific surface area is much higher, which is conducive to cell proliferation. The permeability of TO-P1 is much higher than the other three, but its shear resistance and specific surface area are much lower.

By comparing the normalized values of compressive strength, shear strength, permeability, and specific surface area, it is found that TO-S2 has excellent compressive and shear strength and can provide sound strength effects. Therefore, TO-S2 is suitable for the porous structure of titanium alloy implants subjected to compressive and shear loads.

### 4.3. Establishment of a Porous Titanium Alloy Regulating Model

A certain functional relationship exists between the porosity and mechanical properties of porous structures. To obtain this relationship, the Gibson–Ashby model is established [34]. The Gibson–Ashby model can describe the relationship between the equivalent elastic modulus of the porous structure and the porosity, as shown in Equation (10).

(10) $$\frac{E}{E_{S}}=C{\left(1-P\right)}^{n}$$

In the equation, *E* represents the equivalent elastic modulus of the porous titanium alloy, *E_S_* represents the elastic modulus of the dense titanium alloy, *P* represents the unit cell porosity, and *C* and *n* are constants of the porous structure.

To provide an accurate predictive model for the equivalent elastic modulus and achieve precise control of mechanical properties, the porosity of the TO-S2 porous titanium alloy is divided based on a 5% interval. Porous titanium alloy compression specimens with porosity ranging from 60% to 90% are produced via SLM. The compressive tests are carried out on each specimen three times, and the results are averaged to calculate the equivalent elastic modulus of the TO-S2 porous titanium alloy at different porosity levels.

**Figure 18:**
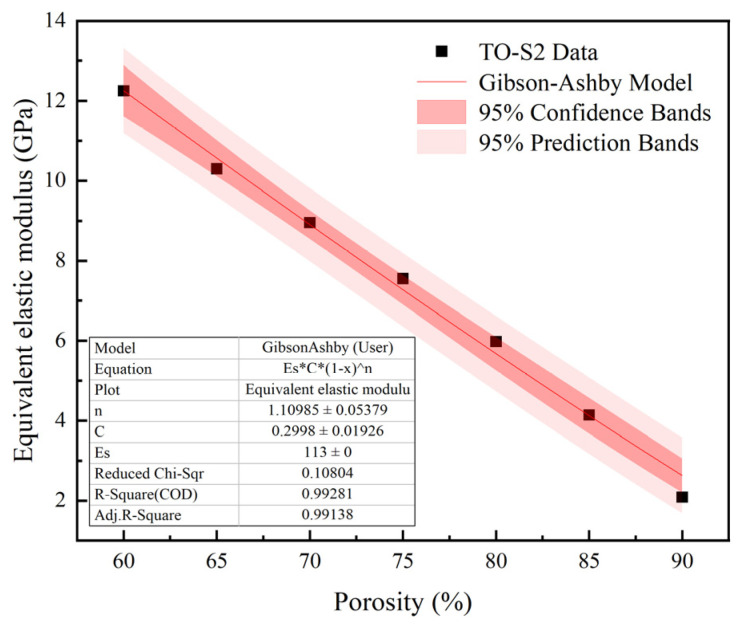
TO-S2 Gibson–Ashby Model.

The Gibson–Ashby model was solved using Origin software to fit a curve. A custom function was created in Origin software: *y* = *E_S_* * *C* * (1 − *x*)*^n^*, where *E_S_* is 113 GPa, *C* and *n* are unknown variables representing the parameters *C* and n in the Gibson–Ashby model. *y* represents the equivalent elastic modulus *E*, and *x* represents the porosity *P*. By fitting the *x* and *y* data within each porosity range, the parameters *C* and *n* were solved as 0.2998 and 1.10985, respectively. The Gibson–Ashby model for TO-S2 was plotted as shown in Figure 18.

By substituting *C* and *n* into Equation (9) and combining it with the mathematical model of porosity and strut diameter in Table 2 of Section 4.1, the corresponding strut diameter parameter *S* for the equivalent elastic modulus *E* is obtained to achieve rapid structure–property matching. The mechanical property regulation model of the TO-S2 porous titanium alloy structure is obtained by combining them, as shown in Equation (11).

(11) $$\sqrt[{n}]{\frac{E}{E_{S}C}}=3.73293S^{2}+1.30624S-0.06768$$

By substituting parameters *C* and n into Equation (5), the equivalent elastic modulus values fitted by the Gibson–Ashby model and the average of three experimental values are shown in Table 9. The equivalent elastic modulus values fitted by the Gibson–Ashby model are within the range of elastic modulus of human cortical bone, which meets the design requirements. The model calculation results are close to the experimental test results, making it an ideal empirical model.

### 4.4. Printing of Trabecular Tibial Implants

Based on the elastic modulus of the segmented range of tibial bone in Table 1 and selecting the TO-S2 structure with the best comprehensive performance, the corresponding strut diameter for the equivalent elastic modulus of the tibial trabecular layers 2 to 4 is calculated using the mechanical property regulation model Equation (6) for porous titanium alloy. This is shown in Table 10. By changing the strut diameter of the porous titanium alloy, the corresponding elastic modulus range can be adjusted to achieve a more accurate match with the mechanical properties of human bones.

**Figure 19:**
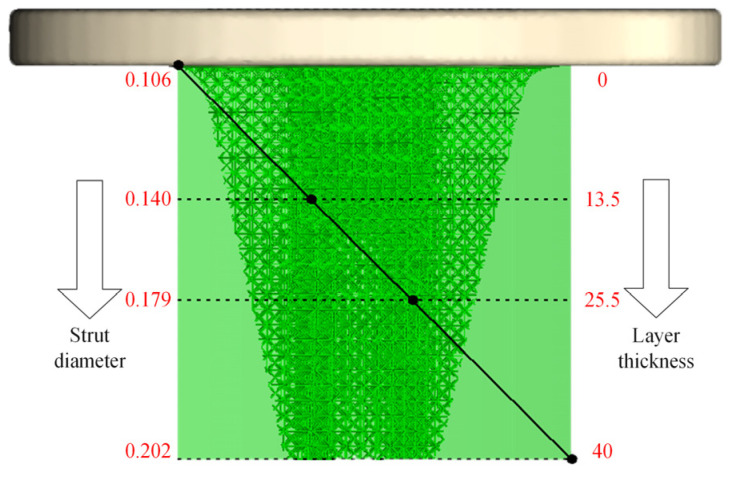
Continuous gradient filling settings.

**Figure 20:**
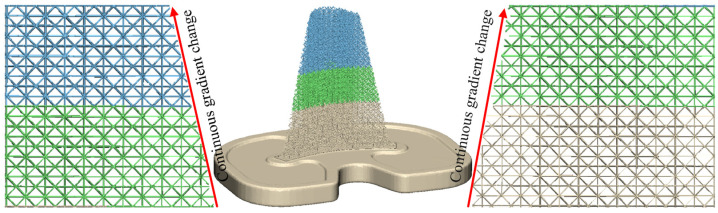
Effect of gradient concatenation.

The elastic modulus of the trabecular bone layer in the tibia bone gradually increases according to the subdivision of the trabecular bone layer. To avoid the step effect at the connection of adjacent trabecular bone layers caused by different porosity forming a porous structure, the TO-S2 porous structure is continuously gradient-filled into the trabecular bone layer of the tibia stem. The continuous gradient filling can better simulate the real structural characteristics of bones, improve the connection effect of adjacent trabecular bone layers, eliminate step effects, and avoid implant failure caused by poor connections. The porous structure’s continuous gradient filling is shown in Figure 19, with the support strut diameter continuously gradient-filled from 0.106 mm to 0.140 mm, 0.179 mm, and 0.202 mm. As shown in Figure 20, the connection effect of continuous gradient filling is good, achieving a complete gradient connection.

**Figure 21:**
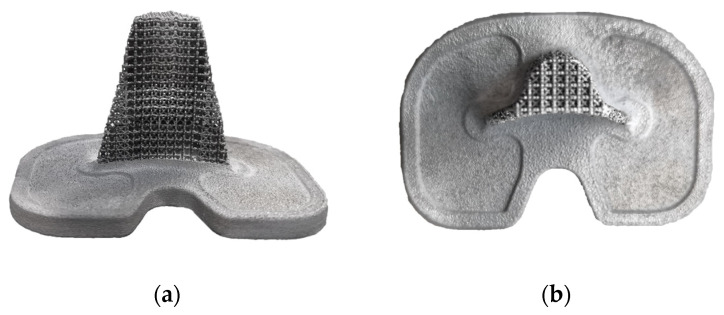
Trabecular tibial implant formation effect: (**a**) front view, (**b**) top view.

After printing personalized porous titanium implants using SLM technology, the implants were sandblasted and ultrasonically cleaned. The resulting trabecular tibial implant is shown in Figure 21.

## 5. Conclusions

The article comprehensively considers the relationship between the loading state of the tibial skeletal joint and the required mechanical performance and designs a novel bone strut tibial implant that matches the human bone structure performance. The conclusion is as follows:

1. A personalized tibial implant solid model was designed based on the loading state of the tibial skeletal joint, and it was placed in the tibial resection area. The elastic modulus of the tibial stem was determined to be between 3.031 and 10.528 GPa.
2. Two types of unit cell structures were designed using topology optimization for compression and shear resistance, respectively (TO-P1, TO-P2, TO-S1, TO-S2), and four common unit cell structures (BCC, FCC, RDC, DCC). A fitting model was established for the relationship between strut diameter and porosity of the unit cell structures.
3. The mechanical and permeability properties of porous titanium alloys were comprehensively compared, and it was determined that the TO-S2 structure had the best overall performance and was most suitable as a trabecular structure for tibial implants.
4. A model was established to regulate the mechanical properties of porous titanium alloys, and it was determined that the strut diameter of porous titanium alloy for tibial implants should be between 0.106 and 0.202 mm, and the tibial stem should be filled with porous titanium alloy. Finally, SLM technology was used to print the trabecular tibial implant with good forming results.

However, this study still has some limitations; there is still some work to be further studied. In the SLM process, the components will experience repeated thermal stress, which may cause residual stress complications. Therefore, it is necessary to consider the effect of residual stress on the compressive strength of porous implants. In this paper, only the shear performance and permeability of the porous structure are simulated and analyzed, and the porous structure can be further studied experimentally. The biomechanical analysis of trabecular tibial implants after implantation can be carried out in the simulation software and then combined with SLM-formed porous titanium alloy implants for experimental research, which provides a good guarantee for practical medical applications.

## Figures and Tables

**Table 1:** Range of partition for tibial trabecular subregions.

Trabecular Layer of Bone	Threshold Range/Hu	Apparent Density *ρ*/(g/cm^3^)	Elastic Modulus E/GPa
Layer 1	500.53–565.27	1.056–1.179	2.329–3.031
Layer 2	565.27–682.63	1.179–1.402	3.031–5.287
Layer 3	682.63–796.84	1.402–1.619	5.287–8.439
Layer 4	796.84–916.78	1.619–1.733	8.439–10.528
Layer 5	916.78–1010.40	1.733–1.805	10.528–12.017

**Table 2:** Topology optimization porous structure name.

Structure Type	Abbreviation of Structure Type
Topology Optimization-Pressure-1	TO-P1
Topology Optimization-Pressure-2	TO-P2
Topology optimization-Shear-1	TO-S2
Topology optimization-Shear-2	TO-S2

**Table 3:** Geometric parameters of different types of topological cells.

	P/%	90	85	80	75	70	65	60
TO-P1	S/mm A/mm	0.222 0.78	0.276 0.73	0.325 0.68	0.37 0.63	0.412 0.59	0.452 0.55	0.49 0.51
TO-P2	S/mm A/mm	0.14 0.567	0.168 0.539	0.198 0.509	0.225 0.482	0.252 0.455	0.279 0.428	0.3 0.407
TO-S1	S/mm A/mm	0.125 0.87	0.157 0.843	0.185 0.815	0.21 0.79	0.235 0.765	0.257 0.743	0.28 0.72
TO-S2	S/mm A/mm	0.1 0.858	0.123 0.826	0.145 0.794	0.165 0.766	0.185 0.738	0.202 0.714	0.22 0.688

**Table 4:** Geometric parameters of four kinds of ordinary basic unit cells.

	P/%	90	85	80	75	70	65	60
BCC	S/mm A/mm	0.145 0.56	0.182 0.52	0.213 0.49	0.242 0.46	0.27 0.43	0.296 0.41	0.32 0.38
RDC	S/mm A/mm	0.1 0.6	0.129 0.58	0.155 0.55	0.173 0.53	0.19 0.51	0.212 0.5	0.23 0.48
DCC	S/mm A/mm	0.13 0.57	0.167 0.54	0.2 0.5	0.225 0.48	0.25 0.45	0.279 0.428	0.3 0.4
FCC	S/mm A/mm	0.132 0.868	0.165 0.835	0.193 0.807	0.22 0.78	0.245 0.755	0.27 0.73	0.293 0.707

**Table 5:** Fitting equation for unit cell structures.

Monoclinic Structure	Fitting Equation	Correlation Coefficient R^2^
TO-P1	P = 1.05271 − 0.49073S − 0.88485S^2^	0.99999
TO-P2	P = 1.09461 − 1.19359S − 1.48474S^2^	0.9993
TO-S1	P = 1.08954 − 1.21685S − 1.9013S^2^	0.99984
TO-S2	P = 1.06768 − 1.30624S − 3.73293S^2^	0.99987
BCC	P = 1.04546 − 0.67328S − 2.24506S^2^	0.99992
RDC	P = 1.02176 − 0.7152S − 4.88784S^2^	0.99963
DCC	P = 1.02005 − 0.54815S − 2.83886S^2^	0.99945
FCC	P = 1.06386 − 0.94786S − 2.17198S^2^	0.99988

**Table 6:** Ti6Al4V alloy material properties.

Elastic Modulus	Yield Strength	Density	Poisson’s Ratio
113 GPa	890 MPa	4.43 g/cm^3^	0.342

**Table 7:** Specific surface area of porous structures (δ/mm^−1^).

Porosity	TO-P1	TO-P2	TO-S1	TO-S2
60%	2.5808	3.824	4.4124	5.6084
70%	2.4378	3.773	4.2336	5.4496
80%	2.147	3.4605	3.7966	4.8854
90%	1.5455	2.8233	3.0264	3.8453

**Table 8:** Permeability coefficients of different porous structures in human bone in the literature.

Porous Structures Type	Porosity (P/%)	Permeability Coefficient (K/10^−9^m^2^)	
Regular CAD	60–90	1–25	Ali [30]
TPMS	75.1–88.8	0.29–3.91	Ma [31]
Micro-CT	78–82	0.75–1.74	Baino [32]
Human bone	-	0.027–20	Nauman [33]
TO-P1	60–90	2.05–13.49	This paper
TO-P2	60–90	1.43–7.1	
TO-S1	60–90	1.45–7.2	
TO-S2	60–90	1.54–7.36	

**Table 9:** Equivalent elastic modulus from experiment and fitting/GPa.

Structure Type	Porosity	Experimental Test Value			Mean	Gibson–Ashby Fitting Value
**TO-S2**	60%	12.266	12.256	12.231	12.251	12.253
	65%	10.277	10.286	10.331	10.298	10.566
	70%	9.138	8.809	8.921	8.956	8.904
	75%	7.713	7.553	7.390	7.552	7.273
	80%	6.158	5.776	6.015	5.983	5.678
	85%	4.051	4.202	4.173	4.142	4.126
	90%	2.014	2.135	2.115	2.088	2.631

**Table 10:** Elastic modulus and strut diameter for different trabecular bone layers.

Trabecular Layer	Elastic Modulus (E/GPa)	Strut Diameter (S/mm)
Layer 2	3.031–5.287	0.106–0.140
Layer 3	5.287–8.439	0.140–0.179
Layer 4	8.439–10.528	0.179–0.202

## Data Availability

Not applicable.
